# SHORT-TERM MRI EVALUATION OF CAPSULOLABRAL REPAIR IN ATHLETES WITH ANTERIOR GLENOHUMERAL INSTABILITY: CORRELATION WITH CLINICAL OUTCOMES

**DOI:** 10.1590/1413-785220253305e287110

**Published:** 2025-09-22

**Authors:** Bernardo Barcellos Terra, Tannous Jorge Sassine, Andre Aihara, Paulo Santoro Belangero, Alberto De Castro Pochini, Benno Ejnisman

**Affiliations:** 1 Santa Casa de Vitoria, Espirito Santo, ES, Brazil.; 2 Universidade Federal de Sao Paulo (UNIFESP), Sao Paulo, SP, Brazil.

**Keywords:** Shoulder Joint, Magnetic Resonance Imaging, Shoulder Injuries, Shoulder Dislocation, Articulação do Ombro, Imagem por Ressonância Magnética, Luxação de Ombro

## Abstract

**Objective:**

The aim of this study was to evaluate capsulolabral repair in athletes with traumatic anterior glenohumeral instability using magnetic resonance imaging (MRI) and correlate it with clinical and epidemiological data.

**Method:**

A prospective therapeutic clinical study was conducted with 36 athletes undergoing surgical treatment. MRI was performed preoperatively and in the third month postoperatively. The morphology, height, angulation, integrity and density of the repaired capsulolabral tissue were evaluated. Linear and logistic regression models were applied.

**Results:**

A total of 36 athletes were evaluated (mean age 29.64 ± 9.08 years). For all numerical variables (morphology, integrity, angles and heights) the differences were statistically significant, except for the coronal angle and homogeneity. Longer time to surgery or multiple dislocations reduced the improvement in morphology. There were no new episodes of dislocation. In the 3-month radiological evaluation, the integrity of the labrum was present in 97% of the patients, however, all patients still had a heterogeneous labrum.

**Conclusion:**

There are statistically significant differences between the morphology, height and angulation of the labrum between the pre- and postoperative periods of patients operated on for glenohumeral instability. Although the 3-month MRI showed integrity of the labrum in almost all athletes, this tissue still presented altered density even with satisfactory clinical results. ***Level of Evidence III; Prospective Study.***

## RESUMO

**Objetivo:**

O objetivo deste estudo é avaliar por ressonância magnética (RM) o reparo capsulolabral de atletas com instabilidade glenoumeral anterior traumática e correlacionar com dados clínicos e epidemiológicos.

**Método:**

Foi realizado um estudo clínico prospectivo terapêutico com 36 pacientes atletas submetidos ao tratamento cirúrgico. Foi realizado RM no pré-operatório e no terceiro mês de pós-operatório. Foram avaliadas morfologia, altura, angulação, integridade e densidade do tecido capsulolabral reparado. Modelos de regressão linear e logística foram aplicados.

**Resultados:**

Um total de 36 atletas foram avaliados (idade média de 29,64 ± 9,08 anos). Para todas as variáveis numéricas (morfologia, integridade, ângulos e alturas) as diferenças foram estatisticamente significantes, exceto para o ângulo coronal e homogeneidade. Maior tempo para a cirurgia ou múltiplas luxações reduziram a melhora na morfologia. Não houveram novos episódios de luxação. Na avaliação radiológica de 3 meses, a integridade do lábio se fez presente em 97% dos pacientes, no entanto todos os pacientes ainda apresentavam o lábio heterogêneo.

**Conclusão:**

Há diferenças estatisticamente significante entre a morfologia, altura e angulação do lábio entre o pré e pós operatório dos pacientes operados com instabilidade glenoumeral. Apesar da RM de 3 meses mostrar integridade do lábio em quase todos os atletas, esse tecido ainda apresentou densidade alterada mesmo com resultados clínicos satisfatórios. ***Nível de Evidência lll; Estudo Prospectivo.***

## INTRODUCTION

The shoulder is the most unstable joint in the human body, with anterior instability being the most common.^1–3^ The incidence of dislocation can reach 23 cases per 100,000 people/year, being more common in males and Caucasians, with recurrence being related to younger ages at the first episode and the presence of associated bone injuries.^4–8^

Surgical treatment with arthroscopic capsulolabral repair has satisfactory success rates of around 95%.^9^ Return to physical activities is generally permitted after 4–6 months, depending on the demands and type of physical activity. To determine this, we use functional assessments with strength and range of motion tests.^10,11^

Although magnetic resonance imaging (MRI) is a validated method for analyzing labrum morphology, it is not routinely used to study the integrity of capsulolabral surgical repair, as the computed tomography that can be used for assessing bone consolidation after bone blocking.^12^

There is little literature regarding postoperative image assessments in patients with anterior glenohumeral instability undergoing arthroscopic capsulolabral repair, especially regarding the integrity and morphology of the repaired tissue.^13–16^ The aim of this study is to evaluate, using magnetic resonance imaging, the morphology and integrity of the labrum three months after arthroscopic capsulolabral repair in patients who practice physical activities with traumatic anterior glenohumeral instability and to correlate the results with clinical and demographic data from this population.

## MATERIAL AND METHOD

This study was approved by the hospital's ethics committee and approved by Plataforma Brazil, under number 46089221.7.0000.5505. A prospective clinical study was conducted in which 36 athletic patients with traumatic anterior instability underwent arthroscopic surgery and were prospectively followed up from January 2021 to December 2022. All patients underwent magnetic resonance imaging (MRI) in the preoperative period and in the third month after surgery.

The epidemiological characteristics of the patients are shown in Table 1.

**Table 1:** Epidemiological characteristics of patients included in the study.

Characteristics of patients	Measures
Age (years)	29.64 ± 9.08
**Sex**	
male	32 (88.89%)
female	4 (11.11%)
**Side**	
right	21 (58.33%)
left	15 (41.67%)
**Time of injury**	
≤ 90 days	15 (41.67%)
> 90 days	21 (58.33%)
**Number of episodes**	
Unique	12(33.33%)
Multiples	24 (66.67%)
**Sport type (Neutral)**	
Pull	12 (33.33%)
Neutral	9 (25.00%)
Neutral	9 (25.00%)
Neutral	9 (25.00%)
Neutral	9 (25.00%)
Neutral	9 (25.00%)

The inclusion criteria were athletic patients with: (1) traumatic anterior dislocation (2) positive physical examination for anterior instability (3) presence of anterior and inferior capsulolabral injury on preoperative magnetic resonance imaging. The exclusion criteria were: (1) bone lesion in the glenoid > 20%, (2) off-track lesion, (3) associated rotator cuff lesion, (4) associated posterior labral lesion, (5) HAGH lesion, (6) multidirectional or posterior instability by clinical evaluation, (7) generalized ligament laxity (Beighton criterion > 4, sulcus test >2+), (8) advanced osteoarthritis (Samilson and Pietro grade 2 or 3), (9) patients who did not agree to participate in the study (TCLE). The exclusion criteria were: associated injuries found during surgery (such as rotator cuff injury and posterior labral injury) and loss of patient follow-up.^17,18^

Data from preoperative MRI and MRI 3 months after surgery were analyzed. Clinical outcomes such as range of motion, new episodes of dislocation, apreension rate, and functional scores (EROE, ROWE, and VAS) were assessed at 3 months postoperatively. The EROE score (Athlete Shoulder Outcome Rating Scale) is a score based on objective criteria (range of motion) and subjective criteria (pain, strength/resistance, intensity, athletic performance), totaling 100 points. A score above 90 is considered excellent, 70-89 is good, 50-69 is fair, and below 50 is poor.^19,20^

All patients included were athletes, according to Araújo and Scharhag.^21^ The types of sports were categorized according to Allain et al. into three groups: neutral sports (no collision or throwing), collision/contact sports, and throwing sports.^22^

**Figure 1:**
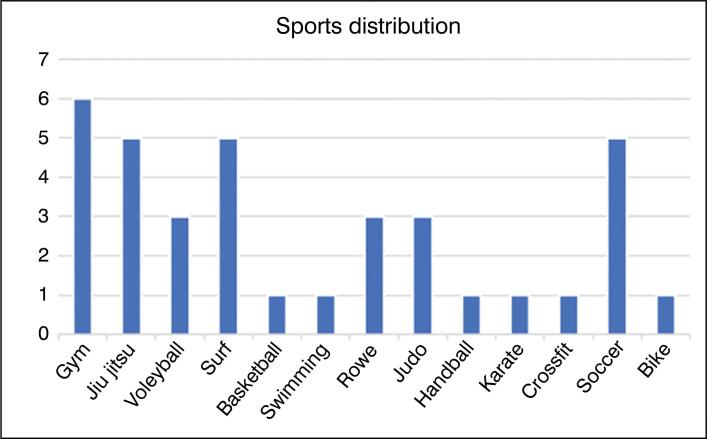
Distribution of types of sports practiced by athletes (frequency x type of sport).

These athletes had the following characteristics: average age 29.6 ± 9.08 years, 32 (88.9%) were male, 21 (58.33%) had right shoulder dislocation, 15 athletes (41.67%) had an injury time of up to 90 days in relation to the first episode, and 24 (66.67%) athletes had multiple episodes of dislocation. With regard to the type of sport, 12 athletes (33.33%) practiced throwing sports, 15 (41.67%) practiced contact sports, and 27 (75%) were amateurs. (Figure 1)

### Surgical procedure

All patients underwent arthroscopic surgery by the same surgeon in the lateral decubitus position and were examined under anesthesia, observing the inferior, anterior, and posterior translation.^8^ The biocomposite anchors used were 3.0 mm double-loaded and knotted (SututeTak, Arthrex®, Naples, USA), with the stitches performed in a simple configuration. The average number of anchors used was 3.22 ± 0.58.

### Post-operative protocol

After the procedure, the operated shoulder was placed in a simple sling and left for approximately 6 weeks. In the third week, passive movements were initiated, avoiding abduction and lateral rotation greater than thirty degrees until the sixth week. At 10 weeks, strengthening began, and at 3 months, a return to activities was permitted. Return to contact or collision sports only after 6 months.

### Image evaluation

Magnetic resonance imaging evaluations of the lip were performed preoperatively and 3 months postoperatively, according to the established and validated protocol of Yoo et al.^23^ applied in several studies. A 1.5 T magnetic resonance imaging (MRI) scanner (GE Signa Explore; GE Healthcare Medical SystemÒ, Boston, USA) with a dedicated shoulder coil was used in all patients. All measurements were performed on a communication and image archiving system monitor (PACS) using the RadiAnt program®.

**Figure 2:**
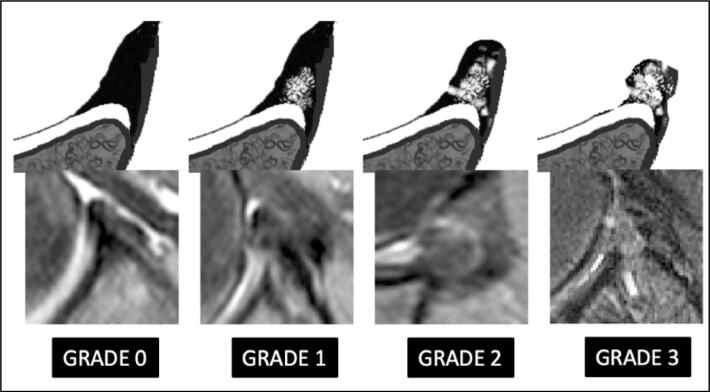
Randelli classification for lip morphology.

The following characteristics analyzed by magnetic resonance imaging (MRI) were considered outcome variables in the study: morphology, axial height, coronal height, axial angle, and coronal angle of the anterior inferior labrum of the glenoid. The morphology of the labrum was measured in the axial section at T2 and graded from 0° to III° according to the descriptive grading published by Randelli et al.^24^ (Figure 2)

**Figure 3:**
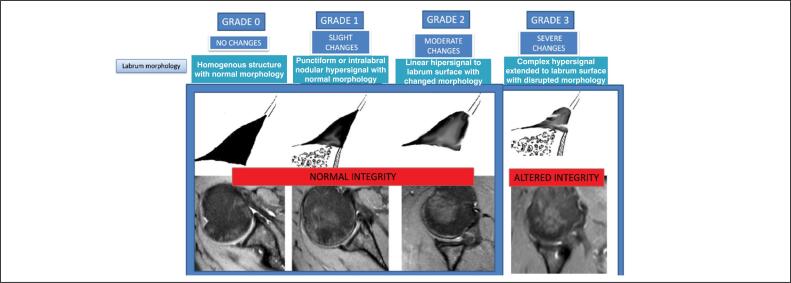
Randelli classification. Intact lip: grades 0, 1, and 2. Lip not fully formed: grade 3.

**Figure 4:**
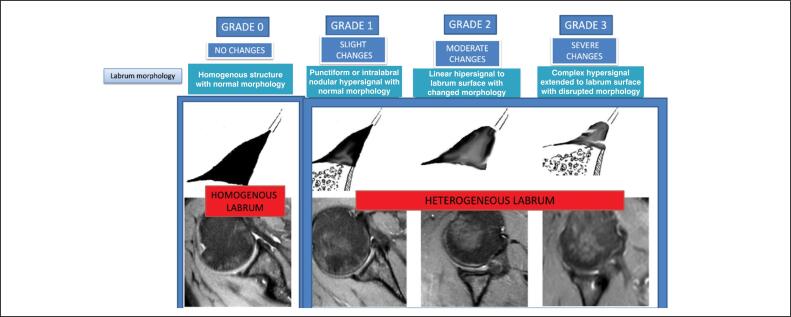
Randelli classification. Homogeneous lip: grade 0. Heterogeneous lip (hypersignal): grade 1, 2, and 3.

Two other outcome variables were lip integrity and homogeneity, both defined based on morphology, using Randelli's classification as a parameter. Integrity was defined as follows: the repair is intact if the morphology is equal to 0, 1, or 2; and not intact if equal to 3 or if there is a continuity solution between the lip and the glenoid (Figure 3). Homogeneity was defined as morphology equal to 0; and heterogeneous if equal to 1, 2, or 3. (Figure 4)

**Figure 5:**
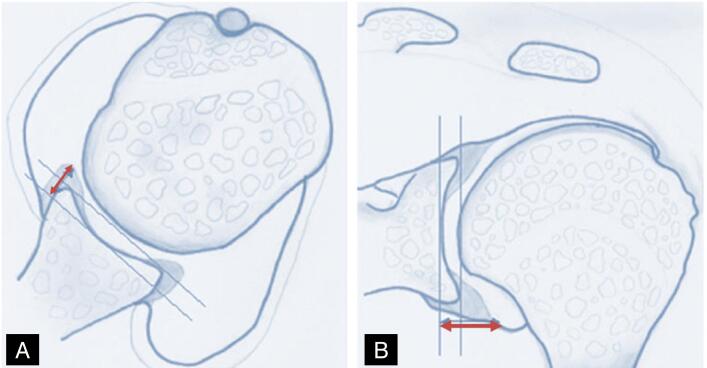
Drawing illustrating method for measuring lip height, axial and coronal sections.

**Figure 6:**
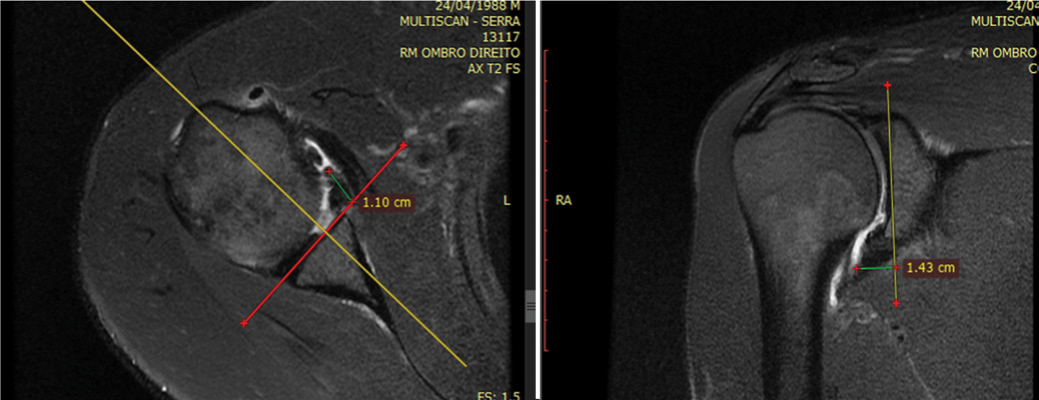
Method for measuring lip height, axial and coronal cuts, using magnetic resonance imaging.

**Figure 7:**
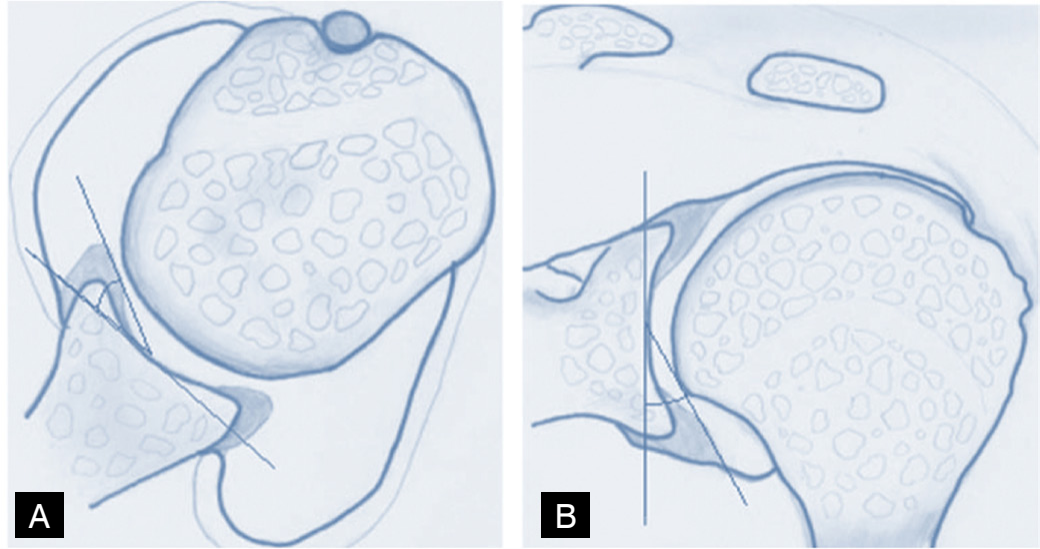
Drawing illustrating the method for measuring lip angulation (slope), axial and coronal sections.

**Figure 8:**
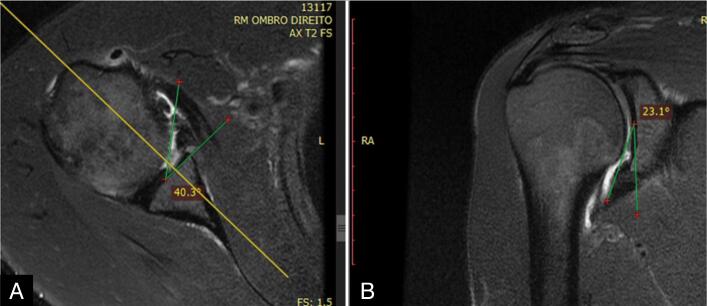
Method for measuring lip angulation, axial section (A) and coronal section (B), on magnetic resonance imaging.

The parameters (morphology, homogeneity, height, and angle) were measured in the axial and oblique coronal images in the anteroinferior portion of the glenoid, immediately above the lowest anchor, thus avoiding the site of the lip where the suture was located so as not to be interpreted as a lesion. Height was defined as the distance (in millimeters) between the lowest portion of the glenoid and the maximum height of the lip tip (Figures 5 and 6). Angulation was defined as the angle formed by a tangential line drawn from the deepest portion of the glenoid (center of the glenoid) to the tip of the highest point of the lip. (Figures 7 and 8)

The measurements were taken at a single point in time by a radiologist who monitored and performed all examinations in consultation with the shoulder surgeon.

### Statistical analysis

A descriptive analysis of the data was performed using the exposure variables. Categorical data are expressed as absolute numbers and percentages, while continuous data are presented as mean ± standard deviation.

For each outcome variable related to MRI, a preoperative comparison was performed and another comparison was performed after 3 months to verify whether there was a statistical difference between the MRI measurements. The nonparametric Wilcoxon-Mann-Whitney test was used for numerical variables, and the McNemar test was used for integrity and homogeneity, which are categorical variables. The power analysis of the test was performed using simulations. Clinically different results were considered, such as improvement in the functional score based on previously published studies, changes of at least 2 mm in axial/coronal height,5° in axial/coronal angle, and 1 point in morphology.^12–14,24,25^ In the simulations, sample standard deviations, a sample size equal to 36, and a significance level of 0.05 were used. For all outcomes, the estimated test power was at least 0.9.

Linear regression models were applied to morphological characteristics, axial height, coronal height, axial angle, coronal angle, elevation, lateral rotation, EROE, ROWE, and VAS scores. Logistic regression models were used for the variables integrity, homogeneity, and medial rotation.

In all tests, a significance level of 0.05 was considered.

## RESULTS

No patient in our prospective series presented a new episode of dislocation or sensation of subluxation during the first three months after surgery. With regard to the range of motion and functional scores in the third postoperative month, the results are shown in Table 2, with a mean EROE of 74.42 ± 12.39, ROWE 82.89 ± 13.72, and VAS 1.89 ± 1.89.

**Table 2:** Clinical data 3 months postoperative

Characteristics	Measures
Elevating the arm	172.2 ± 11.2
Lateral rotation	60.03 ± 18.70
medial rotation (L1)	11 (30.56%)
Medial rotation (L2)	1 (2.78%)
Medial rotation (L5)	1 (2.78%)
Medial rotation (T8)	2 (5.56%)
Medial rotation (T10)	8 (22.22%)
Medial rotation (T11)	2 (5.56%)
Medial rotation (T12)	11 (30.56%)
HEROE	74.42 ± 12.39
ROWE	82.89 ± 13.72
VAS	1.89 ± 1.89

The data regarding the variables measured in the preoperative MRI and at 3 months are shown in Table 3. Except for the coronal angle variable, differences were statistically significant (p-value < 0.05) in all numerical outcome variables.

**Table 3:** Comparison of MRI outcome variables in preoperative and with 3 months.

Moments	Morphology	Alt Axial	Alt Cor	Ang Axial	Ang Cor
Pre-OP	2.58 ± 0.55	8.30 ± 2.79	9.26 ± 2.41	25.8 ± 7.32	23.7 ± 5.31
3 months post-op	1.50 ± 0.56	9.80 ± 2.31	10.4 ± 1.96	29.0 ± 6.45	24.6 ± 4.22
P-value	< 0.001	0,009	0,012	0,012	0,245

OP = operational, p-value for the Wilcoxon-Mann-Whitney test

When evaluating the outcome, there was a significant difference between the proportions of patients with capsulolabral repair integrity in the preoperative and postoperative periods (3 months). With regard to homogeneity, no difference was observed. (Table 4) The variables axial height, coronal height, and axial angle were not related to any of the exposure variables (age, time from injury to surgery, number of episodes, type of sport).

**Table 4:** Comparison for integrity and homogeneity variables

Moments	Integrity	Homogeneity
Pre-OP	14 (38.89%)	0 (0.00%)
3 months post-OP	35 (97.22%)	0 (0.00%)
P-value	< 0.001	1

P-value of the McNemar test.

The association between exposure variables and the difference between pre- and postoperative morphology (called improvement in morphology) was also analyzed. This difference (improvement) was associated with patient age, multiple episodes of dislocation, and time since injury. (Table 5)

**Table 5:** Regression model for difference (morphology pre-morphological post-3months).

Parameter	Estimative	Standard Error	P-value
intercept	2.1848	0.3743	< 0.001
Age	-0.0305	0.0108	0.0079
Multiple episodes	-0.4743	0.2124	0.0327
time of injury (≤90)	0,2857	0,2072	0,1776

R^2^ = 0.3203.

Next, we analyzed the association between exposure variables and the difference between pre- and postoperative coronal angles (improvement in angle). This difference (improvement) was associated with the variables time since injury and occurrence of multiple episodes of dislocation. (Table 6)

**Table 6:** Regression model for difference (3 months coronal angle - pre coronal angle).

Parameter	Estimate	Standard Error	P-value
intercept	-3.636	1.817	0.0537
Multiple episodes	3.68	1.829	0.0524
time of injury (≤90)	5,079	1,749	0,0065

R^2^ = 0.2238.

It was not possible to verify whether there were associations between the exposure variables and the following outcome variables: postoperative shoulder integrity, preoperative and postoperative shoulder homogeneity. This occurs because there is no variability in the results.

**Figure 9:**
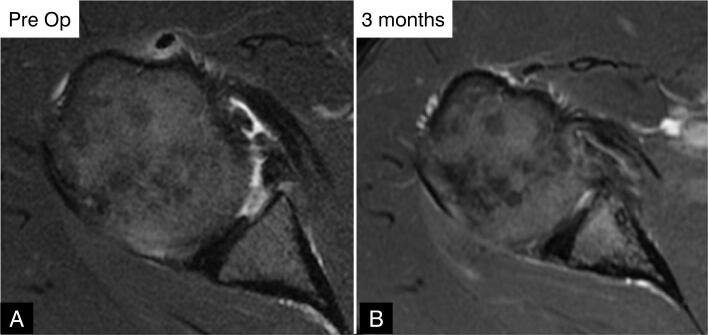
Axial MRI scan of the left shoulder. (A) Preoperative image showing a detached, incomplete labrum and (B) 3 months postoperatively showing an intact but heterogeneous labrum (grade I nodular hypersignal).

**Figure 10:**
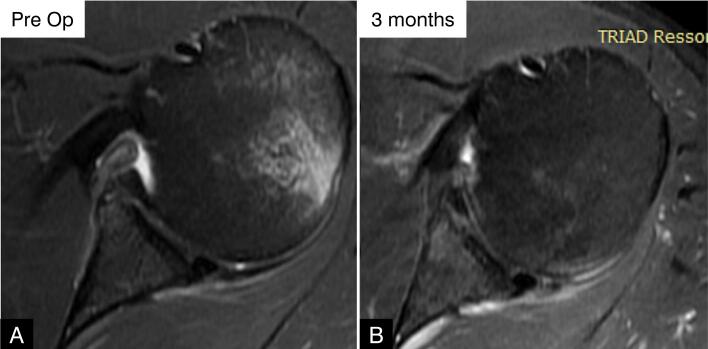
Axial MRI scan of the right shoulder. (A) Preoperative image showing a detached, incomplete labrum and (B) 3 months postoperatively showing an intact but heterogeneous labrum (grade II linear hypersignal).

Taking into account the integrity and density of the labial tissue three months after surgery, 97% of athletes showed repair integrity; however, all still had increased (heterogeneous) signs on MRI evaluation. (Figures 9 and 10)

## DISCUSSION

This study provides evidence that there are statistically significant differences between the preoperative period and 3 months postoperatively for magnetic resonance imaging measurements in athletes who underwent surgery due to shoulder instability. Even with good clinical results (range of motion, functional scores), the repaired lip tissue was heterogeneous in all cases. Athletes with a single episode and an injury duration of up to 3 months showed greater improvement in morphology (integrity).

Lee et al.^20^ evaluated 50 patients with anterior shoulder instability who underwent surgery and underwent computed arthrotomography at 3 months and 12 months postoperatively. The group that presented postoperative apprehension had a higher capsular volume fraction when compared to the group that did not present apprehension. The height of the lip (bumper) measured by early and late arthrotomography showed no statistically significant difference between the two groups. In our study, we used magnetic resonance imaging without contrast due to lower risks of adverse effects. When we assessed the height of the lip in the 3-month MRI, we observed a statistically significant difference compared to the preoperative MRI. However, the labial height in the axial section did not correlate with any exposure variable, whereas the patient's preoperative coronal angle is associated with the duration of the injury and the occurrence of multiple episodes of dislocation. Patients with shorter injury time presented better morphology and better coronal angle gain at 3 months.

Lobo et al.^21^ in their study of 51 patients undergoing arthroscopic treatment for shoulder instability analyzed whether there were differences in functional and imaging results regarding the type of anchor (knotted or knotless) used in arthroscopic treatment of anterior shoulder instability. It concluded that clinical, radiographic, and recurrence results were similar at 24 months of follow-up in both groups. In our study, we used only anchors with knots and observed that the postoperative measurements at 3 months in relation to the parameters analyzed were statistically significant when compared to the preoperative measurements and that patients with up to 90 days of injury and those who do not practice contact sports tend to have a greater gain in the coronal angle (slope) value.

Some studies have evaluated the magnetic resonance imaging characteristics of anterior cruciate ligament reconstructions in the knee and ankle ligament reconstructions using grafts.^26,27^ Although they are tissues with different healing processes when analyzing the labial tissue, some observations can be made. The literature on animal studies has shown that lower graft signal intensity is correlated with greater strength and superior biomechanical properties of the reconstructed ligament.^28,29^ Lower signal intensity (more homogeneous) on preoperative magnetic resonance imaging of the anterior talofibular ligament of the ankle is associated with better clinical outcomes—particularly a higher rate of return to sports.^30^ Although ACL reconstruction studies do not demonstrate a correlation between graft maturity and clinical outcomes,^3,21^ understanding the periods when the graft may be most fragile allows for modification of rehabilitation exercises and even the return of these athletes to the field. When analyzing our results, in the 3-month MRI, the lip was intact in 97% of the athletes, but all still had a heterogeneous lip.

Correlating the MRI results with clinical findings, we observed that even athletes with good range of motion and satisfactory functional results still had hypersignal in the labial tissue in 100% of cases. Given these data, a more careful analysis should be made when allowing these athletes to resume their physical activities, as this tissue may not yet be fully prepared for high loads. It should also be noted that this elevated signal observed on magnetic resonance imaging at three months should be interpreted with caution when assessing the healing of the capsulolabral repair, so as not to be interpreted as a new lesion or lack of healing of the lip.

There are some limitations to our study. Our follow-up was too short to evaluate complications such as recurrence rate or osteoarthritis, as well as to make more detailed comparisons, due to the absence of a control group. However, this is a prospective study with a specific population of athletes, and we evaluated data that has been little explored in previous studies, such as type of sport, number of dislocation episodes, and characteristics of the glenoid labrum on magnetic resonance imaging. The current findings are also useful in postoperative radiographic evaluation. Understanding the normal evolution of capsulolabral tissue is important, and thus the MRI findings in our study can also be used as a reference for radiologists to determine how capsulolabral tissue should look at different time points after arthroscopic repair.

## CONCLUSION

There are statistically significant differences between the morphology, height, and angulation of the lip between the pre- and postoperative periods in patients with glenohumeral instability who underwent arthroscopic surgical treatment.

Despite good clinical results three months after surgery, magnetic resonance imaging showed heterogeneity of the repaired lip tissue in all athletes.

## References

[1] Hovelius L (1978). Shoulder dislocation in Swedish ice hockey players. Am J Sports Med.

[2] Bacilla P, Field LD, Savoie FH (1997). Arthroscopic Bankart repair in a high demand patient population. Arthroscopy.

[3] Werner AW, Lichtenberg S, Schmitz H, Nikolic A, Habermeyer P (2004). Arthroscopic findings in atraumatic shoulder instability. Arthroscopy.

[4] Terra BB, Ejnisman B, Belangero PS, Figueiredo E, De Nadai A, Ton A (2019). Arthroscopic Treatment of First-Time Shoulder Dislocations in Younger Athletes. Orthop J Sports Med.

[5] Ejnisman B, de Figueiredo EA, Terra BB, Cohen C, Monteiro GC, Pochini Ade C (2012). Management of the treatment of glenohumeral instability in patients with extensive bone defect. BMJ Case Rep.

[6] Terra BB, Ejnisman B, Figueiredo EA, Andreoli CV, Pochini AC, Cohen C (2013). Arthroscopic treatment of glenohumeral instability in soccer goalkeepers. Int J Sports Med.

[7] Figueiredo EA, Belangero PS, Cohen C, Louchard RL, Terra BB, Pochini AC (2016). Rodeo athletes: management of shoulder instability. J Sports Med Phys Fitness.

[8] Ejnisman B, de Figueiredo EA, Terra BB, Cohen C, Monteiro GC, Pochini Ade C (2012). Management of the treatment of glenohumeral instability in patients with extensive bone defect. BMJ Case Rep.

[9] Abrams JS, Savoie FH, Tauro JC, Bradley JP (2002). Recent advances in the evaluation and treatment of shoulder instability: anterior, posterior, and multidirectional. Arthroscopy.

[10] Weber A, Paraparan R, Lam PH, Murrell GAC (2019). Return to Sport at 6 Months After Shoulder Surgery. Orthop J Sports Med.

[11] Wilson KW, Popchak A, Li RT, Kane G, Lin A (2020). Return to sport testing at 6 months after arthroscopic shoulder stabilization reveals residual strength and functional deficits. J Shoulder Elbow Surg.

[12] Zhu M, Young SW, Pinto C, Poon PC (2015). Functional outcome and the structural integrity of arthroscopic Bankart repair: a prospective trial. Shoulder Elbow.

[13] Stein T, Mehling AP, Reck C, Buckup J, Efe T, Hoffmann R (2011). MRI assessment of the structural labrum integrity after Bankart repair using knotless bio-anchors. Knee Surg Sports Traumatol Arthrosc.

[14] Stein T, Buckup J, Mehling AP, Hoffmann R, Efe T, von Eisenhart-Rothe R (2014). Restoration of joint congruency and the glenoidal labrum after arthroscopic revision Bankart repair: a MRI match-paired analysis comparing primary Bankart repair and the uninjured labrum. Arch Orthop Trauma Surg.

[15] Sugimoto H, Suzuki K, Mihara K, Kubota H, Tsutsui H (2002). MR arthrography of shoulders after suture-anchor Bankart repair. Radiology.

[16] Buckup J, Welsch F, Petchennik S, Klug A, Gramlich Y, Hoffmann R (2023). Arthroscopic Bankart repair: how many knotless anchors do we need for anatomic reconstruction of the shoulder?-a prospective randomized controlled study. Int Orthop.

[17] Samilson RL, Prieto V (1983). Dislocation arthropathy of the shoulder. J Bone Joint Surg Am.

[18] Beighton P, Horan F (1969). Orthopaedic aspects of the Ehlers-Danlos syndrome. J Bone Joint Surg Br.

[19] Rowe Cr (1963). The surgical management of recurrent anterior dislocations of the shoulder using a modified bankart procedure. Surg Clin North Am.

[20] Leme L, Saccol M, Barbosa L, Ejnisman B, Faloppa F, Cohen M (2010). Translation, cultural adaptation, and validation of the Athletic Shoul- der Outcome Rating Scale into Portuguese [in Portuguese]. Rev Bras Med.

[21] Araújo CG, Scharhag J (2016). Athlete: a working definition for medical and health sciences research. Scand J Med Sci Sports.

[22] Allain J, Goutallier D, Glorion C (1998). Long-term results of the Latarjet procedure for the treatment of anterior instability of the shoulder. J Bone Joint Surg Am.

[23] Yoo JC, Lee YS, Tae SK, Park JH, Park JW, Ha HC (2008). Magnetic resonance imaging appearance of a repaired capsulolabral complex after arthroscopic bankart repair. Am J Sports Med.

[24] Randelli M, Gambrioli PL, Failoni S GM (2001). Computed tomography and magnetic resonance imaging.

[25] Bock J, Buckup J, Reinig Y, Zimmermann E, Colcuc C, Hoffmann R (2018). The arthroscopic Bankart repair procedure enables complete quantitative labrum restoration in long-term assessments. Knee Surg Sports Traumatol Arthrosc.

[26] Bouguennec N, Robinson J, Douiri A, Graveleau N, Colombet PD (2021). Two-year postoperative MRI appearances of anterior cruciate ligament hamstrings autografts are not correlated with functional outcomes, anterior laxity, or patient age. Bone Jt Open.

[27] Li H, Chen J, Li H, Wu Z, Chen S (2017). MRI-based ACL graft maturity does not predict clinical and functional outcomes during the first year after ACL reconstruction. Knee Surg Sports Traumatol Arthrosc.

[28] Amiel D, Kleiner JB, Roux RD, Harwood FL, Akeson WH (1986). The phenomenon of "ligamentization": anterior cruciate ligament reconstruction with autogenous patellar tendon. J Orthop Res.

[29] Janssen RP, Scheffler SU (2014). Intra-articular remodelling of hamstring tendon grafts after anterior cruciate ligament reconstruction. Knee Surg Sports Traumatol Arthrosc.

[30] de Siqueira DC, Baptista AF, Souza I, Sá KN (2014). Tradução, adaptação cultural, validade e confiabilidade do questionário de classificação do ombro para uso no Brasil [Translation, cultural adaptation, validity and reliability of the shoulder rating questionnaire for use in Brazil]. Rev Bras Reumatol.

